# Supplementary material for: Causal Relationship between Aging and Anorexia Nervosa: A White-Matter-Microstructure-Mediated Mendelian Randomization Analysis
Source: Biomedicines. 2024 Aug 16;12(8):1874. doi: 10.3390/biomedicines12081874 (PMC11351342; doi:10.3390/biomedicines12081874)
Supplement: Supplementary file 1 [file biomedicines-12-01874-s001.zip › Supplementary Figure/Figure S4/Singe SNP plot of MR analysis for TL to ubm-b-1767.pdf]

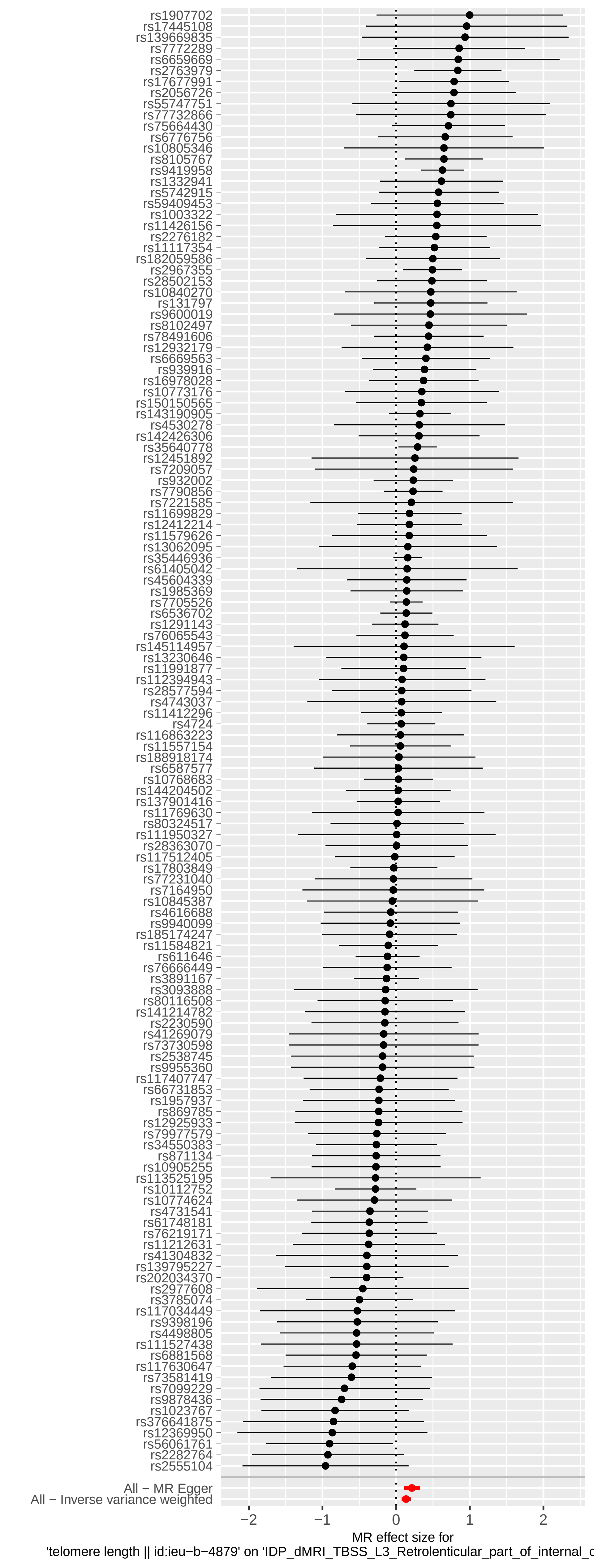
rs1907702
rs17445108
rs139669835
rs7772289
rs6659669
rs2763979
rs17677991
rs2056726
rs55747751
rs77732866
rs75664430
rs6776756
rs10805346
rs8105767
rs9419958
rs1332941
rs5742915
rs59409453
rs1003322
rs11426156
rs2276182
rs11117354
rs182059586
rs2967355
rs28502153
rs10840270
rs131797
rs9600019
rs8102497
rs78491606
rs12932179
rs6669563
rs939916
rs16978028
rs10773176
rs150150565
rs143190905
rs4530278
rs142426306
rs35640778
rs12451892
rs7209057
rs932002
rs7790856
rs7221585
rs11699829
rs12412214
rs11579626
rs13062095
rs35446936
rs61405042
rs45604339
rs1985369
rs7705526
rs6536702
rs1291143
rs76065543
rs145114957
rs13230646
rs11991877
rs112394943
rs28577594
rs4743037
rs11412296
rs4724
rs116863223
rs11557154
rs188918174
rs6587577
rs10768683
rs144204502
rs137901416
rs11769630
rs80324517
rs111950327
rs28363070
rs117512405
rs17803849
rs77231040
rs7164950
rs10845387
rs4616688
rs9940099
rs185174247
rs11584821
rs611646
rs76666449
rs3891167
rs3093888
rs80116508
rs141214782
rs2230590
rs41269079
rs73730598
rs2538745
rs9955360
rs117407747
rs66731853
rs1957937
rs869785
rs12925933
rs79977579
rs34550383
rs871134
rs10905255
rs113525195
rs10112752
rs10774624
rs4731541
rs61748181
rs76219171
rs11212631
rs41304832
rs139795227
rs202034370
rs2977608
rs3785074
rs117034449
rs9398196
rs4498805
rs111527438
rs6881568
rs117630647
rs73581419
rs7099229
rs9878436
rs1023767
rs376641875
rs12369950
rs56061761
rs2282764
rs2555104
All − MR Egger
All − Inverse variance weighted
−2
−1
0
1
2
MR effect size for
'telomere length || id:ieu−b−4879' on 'IDP_dMRI_TBSS_L3_Retrolenticular_part_of_internal_c
